# Effects of a Calorie-Restricted Mediterranean-Style Diet on Plasma Lipids in Hypercholesterolemic South Korean Patients

**DOI:** 10.3390/nu13103393

**Published:** 2021-09-27

**Authors:** Da-Hye Son, Yu-Jin Kwon, Hye Sun Lee, Hyung-Mi Kim, Ji-Won Lee

**Affiliations:** 1 Department of Family Medicine, College of Medicine, Yonsei University, Seoul 03722, Korea; sonda@yuhs.ac (D.-H.S.); digda3@yuhs.ac (Y.-J.K.); 2 Biostatistics Collaboration Unit, Department of Research Affairs, College of Medicine, Yonsei University, Seoul 03722, Korea; hslee1@yuhs.ac; 3 Department of Food and Nutrition, Dongduck Women’s University, Seoul 02748, Korea; veronykim@naver.com

**Keywords:** mediterranean diet, dyslipidemia, cardiovascular risk, insulin resistance, chronic inflammation

## Abstract

The objective of this randomized cross-over trial was to evaluate the short term effects of a calorie-restricted Korean style Mediterranean diet (KMD) versus a calorie-restricted conventional diet on lipid profile and other metabolic parameters in hypercholesterolemic patients. Ninety-two patients with hypercholesterolemia were randomly assigned to two groups and switched to the other group following a 4-week intervention after a 2-week washout period. While participants during KMD intervention period received home delivery of two meals daily except for weekends, those during the control group were advised to consume a conventional diet. Total cholesterol, low-density lipoprotein cholesterol (LDL-C), and high-density lipoprotein cholesterol (HDL-C) significantly decreased in KMD group even after adjusting for age, sex, total energy intake changes, alcohol consumption, smoking status, and physical activity changes (all *p* < 0.05). Anthropometric parameters, white blood cell (WBC), fasting glucose, fasting insulin, HOMA-IR, and fatty liver index (FLI) also significantly decreased after KMD intervention (all *p* < 0.05). In addition, WBC, fasting glucose, total cholesterol, LDL-C and FLI were significantly decreased even after adjusting for weight reduction changes. Calorie-restricted KMD not only helps to treat dyslipidemia by improving the lipid parameters but also has beneficial effects on reducing cardiovascular risk by improving chronic inflammation, insulin resistance, and fatty liver.

## 1. Introduction

Dyslipidemia is a metabolic abnormality defined as elevated low-density lipoprotein cholesterol (LDL-C) and often is combined with low levels of high-density lipoprotein cholesterol (HDL-C) and elevated triglycerides (TG). Dyslipidemia is one of the leading causes of cardiovascular disease (CVD) and is a risk factor that is modifiable by lifestyle management [1]. In Korea, the prevalence of dyslipidemia in adults over 30 years of age increased from 8.0% to 19.9% from 2005 to 2016 [2]. This trend may be related to the recent changes in Korean dietary habits to a western style, characterized by excessive intake of fat, sugar, animal protein, and a low intake of fruits and vegetables [3]. The 2016 European guidelines suggest that adequate changes in diet and lifestyle may prevent approximately 80% of premature CVD mortality [4]. Therefore, many countries recommend Mediterranean diets (MDs) and the Dietary Approaches to Stop Hypertension (DASH) diet as a therapeutic lifestyle modification [3,5].

The MD emphasizes high consumption of olive oil, fruits, root vegetables, legumes, nuts, seeds, and whole grains to provide significant amounts of monounsaturated fatty acids and reduced saturated fatty acid intake. It is also characterized by moderate consumption of red wine with meals, moderate consumption of fish, poultry, dairy products, and low intake of red meat and sweets [6]. Since Ancel Keys proposed the MD model, many studies have shown that the MD has beneficial effects in preventing several diseases, such as CVD, type 2 diabetes, metabolic syndrome, inflammatory disease, and cancer [7,8,9]. Although there is consistent evidence that the MD has beneficial effects on chronic non-communicable diseases, MD intervention studies conducted in non-Mediterranean populations, East Asians in particular, are very limited. Due to the diversity of dietary cultures across countries and races, our research team developed a Korean style Mediterranean diet (KMD) with the ideal ratio of macronutrients to increase life expectancy of Koreans while maintaining the core concept of the MD [10].

The primary aim of this study is to evaluate the short-term effects of a low-calorie KMD compared to a calorie-restricted conventional diet following nutritional advice for Dietary Reference Intakes (DRI) for Koreans in relatively healthy subjects with hypercholesterolemia. We hypothesize that this 4-week low-calorie KMD intervention will reduce cardiovascular risk through lipid reduction and have beneficial effects on other biomarkers, such as inflammatory markers, fatty liver index (FLI), and insulin resistance markers.

## 2. Materials and Methods

### 2.1. Study Participants

Asymptomatic adults with hypercholesterolemia who voluntarily visited the Family Medicine Clinic at Gangnam Severance Hospital, South Korea, were recruited into a protocol approved by the institutional review board and gave written informed consent. Volunteers were eligible if they had at least one of the following criteria: total cholesterol ≥ 200 mg/dL, LDL-C ≥ 130 mg/dL, or TG ≥ 200 mg/dL. Exclusion criteria included a history of cardiovascular disease or stroke, current cancer treatment, uncontrolled hypertension (systolic blood pressure >180 mmHg or diastolic blood pressure >120 mmHg), uncontrolled diabetes or fasting glucose ≥ 200 mg/mL, hepatic disease (aspartate aminotransferase [AST] or alanine aminotransferase [ALT] > 3× institutional upper limit of normal) or renal disease (serum creatinine > 2.0 mg/dL), acute infectious disease (pneumonia, acute gastroenteritis, or urinary tract infection), lipid-lowering medications, other clinical trial medications, vegetarian diet, food allergies, pregnant women, and those who are not able to eat more than 10 MD meals in a row.

**Figure 1:**
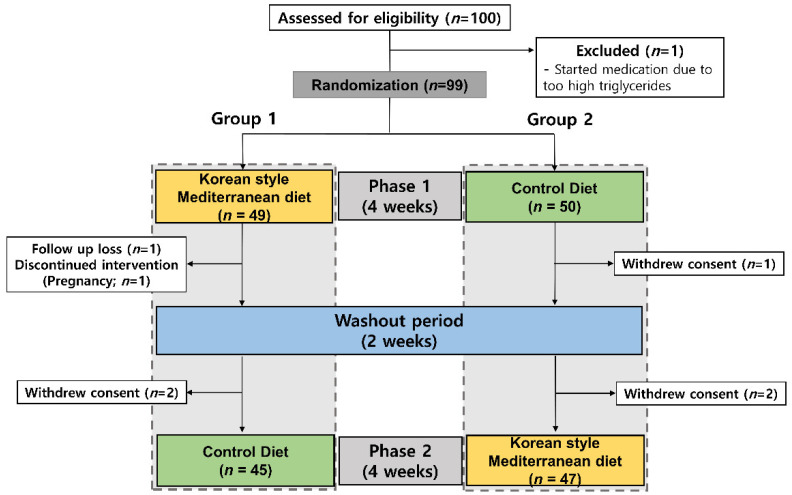
Flow chart of study cohort enrollment, allocation, and completion.

Among 100 participants assessed for eligibility, one patient with triglycerides of 1200 was excluded for starting lipid-lowering medication after an interview with the physician before randomization. Patients consenting to participate in the study were randomized into two groups. Patients in group 1 performed the KMD intervention first, followed by the control diet, and group 2 followed the conventional diet first and then the KMD intervention. After the phase 1 intervention, two patients (one lost to follow-up, one became pregnant) from group 1 and one patient (withdrew consent) from group 2 were excluded. After the phase 2 intervention, two patients from each group were excluded due to withdrawal. A total of 92 patients completed the full 10-week trial (Figure 1).

### 2.2. Study Design

This study was a single-center, randomized, crossover, open-label study conducted at Gangnam Severance Hospital, South Korea, from April 2020 to May 2021. Randomization was based on a random-number table prepared by a biostatistician. After randomization, study participants and researchers who administered the interventions were not blinded, but researchers who assessed outcomes were blinded. Participants were randomized to the KMD or a control diet for 4 weeks, followed by a 2-week washout period and a second 4-week crossover phase (Figure 1).

The study design and experimental protocol were approved by the Institutional Review Board of Gangnam Severance Hospital (IRB number 3-2020-0049) under the Declaration of Helsinki. Written informed consent was obtained from each participant before screening and data collection. This trial was registered at ClinicalTrials.gov (number: NCT04486664).

### 2.3. Dietary Interventions

**Figure A1:**
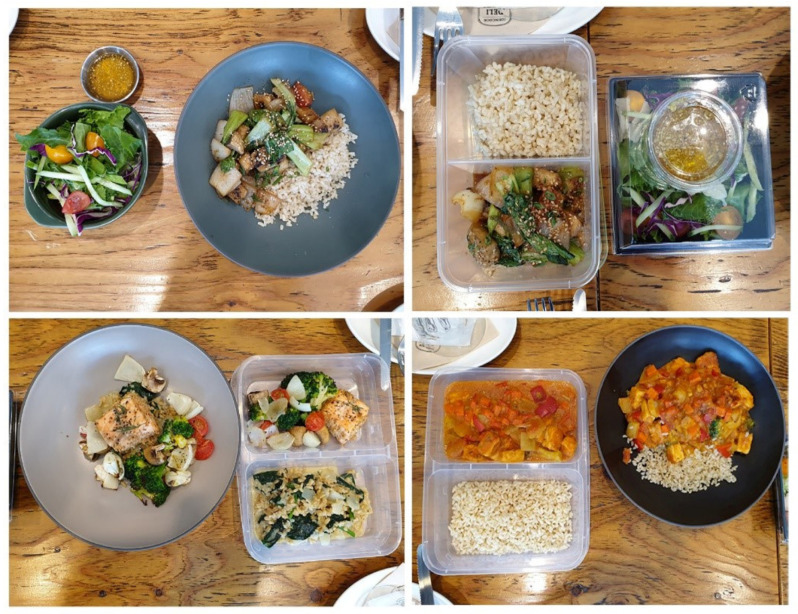
Examples of KMD packages.

The participants allocated to the KMD received home delivery of two meals (lunch and dinner) daily except for weekends during the 4-week intervention period and were directed to eat no fewer than seven meals every week. The meals were prepared by chefs trained on the principles of the MD, under the supervision of professional nutritionists. In addition, the nutritionist provided dietary advice and a leaflet to ensure adherence to the MD pattern for the remainder of the week’s meals not provided with the convenience meal package. Women were instructed to eat salad for breakfast, two MD pattern meals, along with nuts, low-fat milk, and fruits for snacks; men were instructed to eat regular breakfast, two MD pattern meals, and the same snacks as women. The nutritionist assessed compliance and provided feedback during the weekend using the mobile application Kini care. KMD packages were made according to the core concept of the MD and consisted of a carbohydrate: protein: fat ratio of 5:2:3 and had an omega-6 to omega-3 fat ratio of less than 4 to 8 in consideration of Korean dietary habits. In addition, the KMD packages include fewer grains and higher proportions of fish, seafood, and tofu than traditional Korean diets. Two KMD packages per day include 15 g of olive oil (3 servings), 1.5 servings of fruit, 4 servings of vegetables, 1 serving of nuts, and 3.5 servings of fish and meat. Red wine was not included in this analysis. The types of food were substituted by matching the foods using a Korean food exchange list that is similar to the food group classified in the Mediterranean diet pyramid. Examples of the KMD package are presented in Figure A1. The composition of macronutrients was established based on the result of calculating the macronutrient ratio associated with the lowest all-cause mortality from the Korea National Health and Nutrition Examination Survey (KNHANES) linked with causes of death data by our research team [10].

During the control period, participants were advised to consume a conventional diet consisting of 55–65% carbohydrate, 7–20% protein, and 15–30% fat, with 4–10% from omega-6 fat and 1% from omega-3 fat, and no more than 300 mg cholesterol per day, based on the DRI for Koreans 2015. Participants received only dietary training without meal delivery during the control period. Total caloric intake was restricted to 1500 kcal for men and 1300 kcal for women in both the KMD and control diet periods. The same nutritionist gave dietary advice to participants in both groups and all participants had continuous access to the nutritionist for advice and consultation throughout the study. During the 2-week washout period, subjects were instructed to consume their usual diet before the clinical trial. Nutritionists assessed compliance using the mobile application Kini Care, which was developed for nutrition management of patients with metabolic diseases and cancer [11]. This smart phone-based application provides nutritional analysis and calorie analysis based on the Korean food intake and analysis system (K-FIAS), a raw material analysis system. During both the KMD and control diet periods, we recommended that participants do moderate-intensity aerobic activity for 150 min every week.

### 2.4. Measurements and Endpoints

Anthropometric measurements were taken by one qualified provider while the participants were barefoot and wearing light clothes. Body weight (nearest 0.1 kg) was measured along with height (nearest 0.1 cm) using an automatic extensometer (BSM 330; Biospace, Seoul, South Korea). Body mass index (BMI) was calculated as the weight in kilograms divided by the square of the height in meters (kg/m^2^). Body composition, including skeletal muscle mass, fat mass, and fat percentage, was assessed using a bioelectrical impedance analyzer (ACCUNIQ BC720; SELVAS Healthcare Inc., Daejeon, South Korea). Waist circumference was measured at the horizontal plane midway between the lowest ribs and the iliac crest with the participant in a standing position.

**Figure 2:**
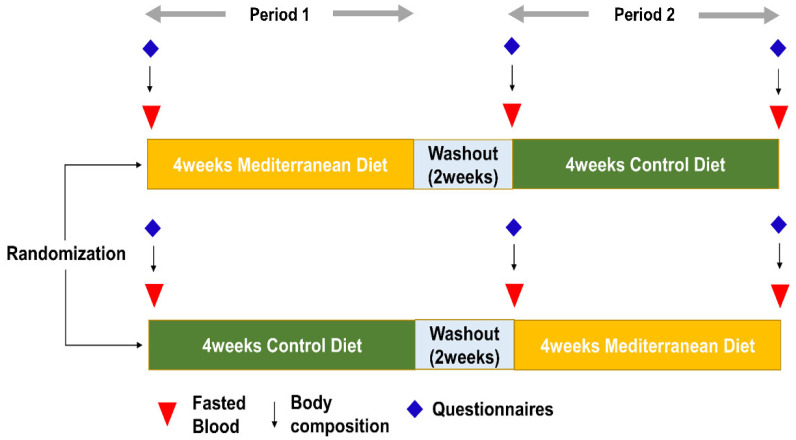
Scheme of study.

Fasting blood samples were collected from an antecubital vein at baseline and at the end of each diet period (Figure 2). White blood cell (WBC) counts were quantified with an XN-9000 hematology analyzer (Sysmex, Lincolnshire, IL, USA). Fasting glucose, high-sensitivity C-reactive protein (hs-CRP), total cholesterol, triglyceride, LDL-C, and HDL-C levels were measured with the ADVIA 1650 Clinical Chemistry system (Siemens Medical Solutions, Tarrytown, NY, USA). Apolipoproteins Al and B were estimated using the Cobas c702 chemistry autoanalyser (Hitachi Co., Tokyo, Japan). Fasting insulin was measured using an electrochemiluminescence immunoassay using an Elecsys 2010 instrument (Roche, Indianapolis, IN, USA). Insulin resistance was estimated using the homeostasis model assessment of insulin resistance (HOMA-IR) method by applying the following formula: HOMA-IR = fasting insulin (μIU/mL) × fasting glucose (mg/dL)/405. FLI was calculated by the following formula [12]:

FLI = (e^0.953 × loge(triglycerides) + 0.139 × BMI + 0.718 × loge(GGT) + 0.053 × waist circumference − 15.745^)/(1 +
e^0.953 × loge(triglycerides) + 0.139 × BMI + 0.718 × loge(GGT) + 0.053 × waistcircumference − 15.745^) × 100.

At baseline and at the end of each diet period, health-related (e.g., physical activity, smoking, and alcohol consumption) and food intake questionnaires were administered. The Godin Leisure-Time Exercise Questionnaire was used to calculate the quantity of physical activity in metabolic equivalent hours per week. The 24-h dietary recall method was used to calculate the intake of total calories and specific nutrients for one day. The degree of adherence to the MD was assessed using the Korean version of the Mediterranean Diet Adherence Screener (K-MEDAS), which was developed by our research group [13].

Safety assessments included evaluations of treatment-emergent adverse events, concomitant medications, vital signs (recorded at each visit), and clinical laboratory measures (recorded at baseline and the end of each diet), including serum creatinine and liver function tests.

The primary efficacy endpoint was a change in serum lipid levels. Secondary endpoints were changes in weight, BMI, waist circumference, WBC count, hs-CRP, glycemic variables (fasting glucose, fasting insulin, HOMA-IR), and FLI.

### 2.5. Statistical Analysis

All statistical analyses were performed using SAS 9.4 (SAS Institute, Cary, NC, USA) and SPSS for windows (version 23.0; SPSS, Chicago, IL, USA). Differences in nutrition components between two groups were analyzed using an independent t-test if the data were normally distributed and a Mann–Whitney U test if not. The fixed effects of MD, period, and sequence were compared using a linear mixed model after adjusting for age, sex, total energy intake changes, alcohol consumption, smoking status, and physical activity variance. The variables with carry-over effect were analyzed using the paired t-test after period 1. *p* < 0.05 was considered statistically significant.

The sample size was calculated according to our primary endpoints. Differences in total cholesterol between a rural diet (MD) and an urban diet were selected as minimum clinically significant values [14]. The sample size was calculated using a two-sided *t*-test with 90% power, a significance level of 0.05 (two-sided), and a dropout rate of 25%.

## 3. Results

We recruited 100 eligible subjects, and 92 subjects completed the full trial (Figure 1). The clinical characteristics are shown in Table 1. Participants who received the KMD showed significantly lower total energy intake, lower intake of trans fat and cholesterol, higher intake of monounsaturated fatty acids (MUFAs) and n-3 polyunsaturated fatty acids (PUFAs), and higher K-MEDAS scores compared with the control diet group (Table 2). There was no significant difference between the KMD and control diets in the absolute amount of fiber; however, the fiber content per 1000 kcal was higher in the MD group (10.2 g/1000 kcal) than that in the control diet (7.7 g/1000 kcal).

**Figure 3:**
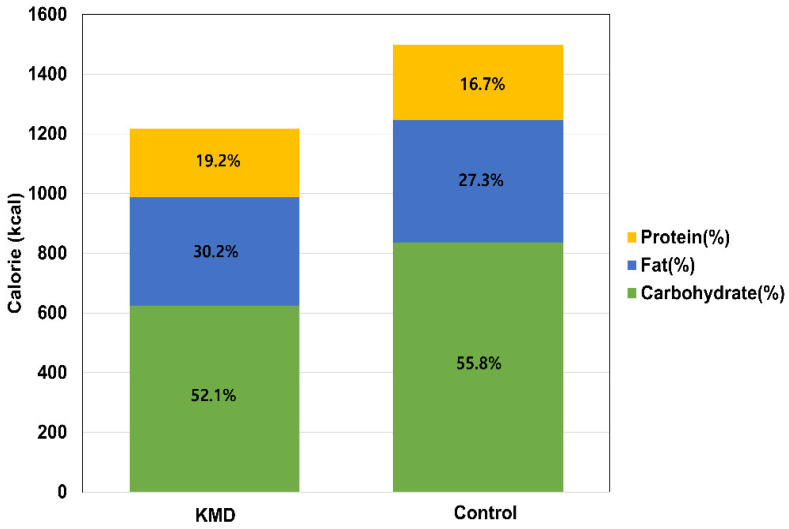
Macronutrient composition of each diet group.

The KMD consisted of a 5:3:2 carbohydrates: fat: protein ratio, whereas the control diet showed a higher proportion of carbohydrates (55.8%) (Figure 3). K-MEDAS was significantly higher in the KMD group than in the control group.

After KMD intervention, total cholesterol, HDL-C, and LDL-C significantly decreased compared to the control diet even after adjusting for age, sex, total energy intake changes, alcohol consumption, smoking status, and changes in physical activity (Table A1). Weight and BMI were decreased in the KMD group compared to the control group after adjusting for confounding variables. Cardiometabolic parameters, including WBC count, fasting glucose, fasting insulin, HOMA-IR, and FLI, also significantly decreased after the KMD period compared to the control diet after adjustment. The actual values of each parameter are reported in Table A2.

**Figure 4:**
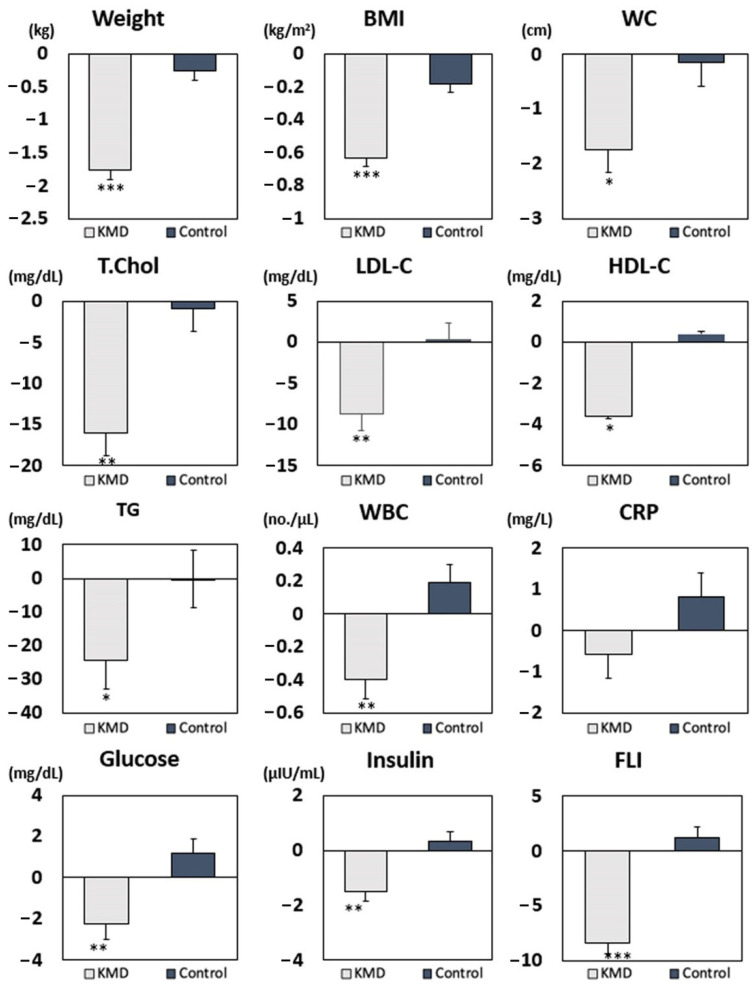
Mean changes in anthropometric and cardiometabolic parameters according to the KMD and control diet. Abbreviations: KMD, Korean style Mediterranean diet; BMI, body mass index; WC, waist circumference; T.chol, total cholesterol; LDL-C, low-density lipoprotein cholesterol; HDL-C, high-density lipoprotein cholesterol TG, triglycerides; WBC, white blood cell; CRP, C-reactive protein; FLI, fatty liver index. * *p*-value < 0.05, ** *p*-value < 0.005, *** *p*-value < 0.0001.

The sequence in which participants consumed each diet did not affect the mean changes in weight, BMI, waist circumference, and metabolic factors, except for mean change in FLI. A significant period effect was shown in the mean changes of total cholesterol, HDL-C, and LDL-C. Therefore, we analyzed these three metabolic parameters only after period 1 (Table A3) and saw a significant decrease in total cholesterol and LDL-C after MD period 1. However, since the amount of change per variable was tremendous, these variations require detailed interpretation and therefore calls for further research. We plan to explore these variables by increasing the study population and subdividing it into groups that can secure more homogeneity. Figure 4 shows that body weight, BMI, waist circumference, and all metabolic parameters significantly decreased in the KMD group in both periods. Furthermore, when we adjusted for weight reduction and added previous confounders, WBC, fasting glucose, total cholesterol, LDL-C, and FLI were still significantly decreased in the KMD group compared with the control group (Table A4). These results suggest that the KMD improves metabolic parameters beyond the improvement seen with weight reduction. Adverse events were not detected during the full clinical trial. Safety was measured using vital signs and clinical laboratory measures (serum creatinine and liver function tests).

## 4. Discussion

In this randomized controlled crossover trial, we found that BMI, lipid profile, glycemic index, WBC count, and FLI decreased after 4 weeks on the calorie-restricted KMD. Further, after adjusting for weight reduction, WBC, fasting glucose, total cholesterol, LDL-C, and FLI still showed statistically significant reductions, so the effect of the KMD is not solely due to weight reduction.

Our study results are consistent with the findings of previous studies examining the beneficial effects of an MD on various cardiometabolic diseases [15,16,17,18]. A randomized trial comparing the short-term effects of an MD versus a low-fat diet reported that a MD with virgin olive oil or mixed nuts resulted in lower blood pressure, improved lipid profile level, decreased insulin resistance, and reduced inflammatory markers compared with a low-fat diet [19]. Esposito et al. reported that an MD improved glycemic control and delayed the need for anti-hyperglycemic drug therapy in patients with newly diagnosed type 2 diabetes [20]. Several studies have reported that the MD or the frequent consumption of olive oil, nuts, or red wine has anti-inflammatory effects [21,22,23,24,25,26,27,28]. A long-term clinical study suggested that MDs and low-carbohydrate diets improved cardiometabolic parameters by reducing hepatic fat content and showed beneficial effects on nonalcoholic fatty liver disease (NAFLD) [29,30]. On the other hand, the decrease in HDL-C after MD is the opposite result to that reported in previous studies; however, it seems to be the effect of calorie restriction. Several previous studies have shown that HDL-C levels are reduced after calorie restriction intervention [31,32]. Nevertheless, since LDL-C is the criterion for initiating medication in patients with dyslipidemia, the result of this study showing significant reductions in LDL-C through KMD is clinically important in terms of delaying the initiation of medication in patients.

However, most of the previous studies investigated adherence to the conceptual guidelines of a MD or provided only the primary components of the MD, such as extra virgin olive oil and nuts, and there were few studies providing Mediterranean style prepared meal packages. Even more, it is not well known whether a MD is useful for East Asians as well as western populations. In our study, hypercholesterolemic patients were provided with convenience meals adhering to the core concepts of the MD with the ideal ratio of macronutrients that could lower the mortality rate of Koreans and improve several cardiometabolic parameters.

The exact mechanisms explaining the favorable effects of adhering to the MD have not been fully elucidated. However, several plausible pathways have been elucidated. Fatty acid composition of a MD is characterized by low cholesterol and saturated fatty acids (SFAs) and high MUFA and PUFA contents [33]. High intake of SFAs increases LDL-C by inhibiting LDL receptor activity and stimulating apolipoprotein B-containing lipoprotein production [34]. However, when SFAs are partially replaced with PUFAs or MUFAs, total and LDL-C levels decrease [35]. The high intake of phytosterols and water-soluble fibers from nuts, seeds, whole grains, vegetables, and fruits may also play a significant role in lowering plasma cholesterol levels. Phytosterols compete with intestinal cholesterol absorption [36] and water-soluble fibers increase the rate of bile excretion, therefore, reducing serum total and LDL-C [37]. In our study, after KMD intervention, mean total cholesterol and LDL-C were decreased by approximately 16 mg/dL and 8 mg/dL, respectively. However, in contrast to the previous study, HDL-C also decreased after KMD intervention. We assume that the effect of weight reduction after dietary intervention led to conflicting results in the HDL-C level. After adjusting for weight reduction, the group difference in mean HDL-C between the KMD and control diet groups disappeared.

Low-grade inflammation is implicated in the development of chronic metabolic disorders, such as type 2 diabetes, NAFLD, and CVD [38]. The clinical benefits of the MD are suggested to be mostly from the anti-inflammatory and antioxidant capacities of various component nutrients [39]. A meta-analysis, including 17 randomized controlled trials, provided evidence that the MD decreases the levels of inflammatory markers such as CRP, interleukin-6, and adhesion molecules [40]. In line with this, we observed that the WBC count significantly decreased and CRP levels decreased marginally after the KMD intervention.

Current evidence indicates that intake of a high omega-6 to omega-3 ratio is associated with a pro-inflammatory response and promotes the development of many chronic diseases [41]. Although the optimal level varies with the disease under consideration, the World Health Organization (WHO) recommends an omega-6 to omega-3 ratio of 5/8, which should form 1–2% of total energy intake per day [42]. In this context, we provided Korean style Mediterranean meal packages with omega-6 to 3 ratios of less than 4/8 in consideration of Korean dietary habits and the WHO guidelines.

The MD has desirable attributes for glycemic control and reducing hepatic fat accumulation, including low-glycemic–index carbohydrates, moderate-to-high content of vegetables, and a moderate fat content [43]. Low-glycemic–index meals improve postprandial glycemia, possibly by reducing non-esterified fatty acid concentrations [44] and hepatic triacylglycerols [45]. Omega-3 PUFA regulates hepatic gene expression of the peroxisome proliferator-activated receptor alpha (PPAR alpha), and omega-9 fatty acids exert important effects in regulating gene expression related to insulin sensitivity [46] as well as lipid and glucose metabolism [47]. In addition, dietary antioxidant intake leads to modulation of insulin resistance and inflammatory cytokine generation [48]. Our results further support the beneficial effects of a MD with favorable changes in fasting glucose, insulin, HOMA-IR, and FLI.

The present study has several limitations. First, the duration of the intervention (4 weeks) was relatively short. Second, the sample size was small compared to previous studies. Thus, further large-scale and long-term investigations are required to evaluate the possible consequences on metabolic parameters. Third, the study was performed in subjects with hypercholesterolemia; therefore, the results may not be generalizable to the overall population. Fourth, since blood samples were not collected after the 2-week washout period, baseline values could differ from those at visit 2. To minimize this limitation, we analyzed sequence and period *p*-values. In addition, we applied calorie restriction along with MD; as such, it is difficult to conclude that MD alone contributed to the results of this study, even after adjusting for calorie intake changes. Finally, whether the clinical effects of the MD are due to the overall dietary pattern or are the sum of effects of individual nutritional components has been controversial. Therefore, additional large-scale, long-term cohort studies or experimental studies are needed to clarify the exact physiological mechanism of the MD.

However, despite these limitations, the present study has several strengths, including an adequately powered sample size and its design as a randomized controlled crossover trial. Moreover, whereas most MD randomized controlled trials evaluated MD adherence based on a self-reporting questionnaire, we provided the same MD style meals to all participants, and nutritionists monitored daily food intake and assessed compliance using a mobile application during the intervention period to minimize recall bias. Finally, this study achieved high compliance rates of more than 90%.

## 5. Conclusions

In conclusion, the results of our study suggest that the calorie-restricted MD not only helps to treat dyslipidemia by improving the lipid profile in hypercholesterolemia patients but also has beneficial effects on reducing CVD risk by improving chronic inflammation, hyperglycemia, and fatty liver disease independently of individual energy intake, physical activity, and weight reduction changes.

## Figures and Tables

**Table 1:** Baseline characteristics of study participants before intervention.

Characteristic	Total (*n* = 92)
Age (years)	45.0 ± 9.5
Male (%)	35 (38%)
Anthropometric parameters	
Weight (kg)	67.6 ± 13.1
BMI (kg/m^2^)	24.8 ± 3.8
Waist circumference (cm)	84.7 ± 10.3
Metabolic parameters	
WBC count (no./μL)	5.6 ± 1.3
Log transformed CRP (mg/L)	−0.6 ± 1.1
Fasting glucose (mg/dL)	100.7 ± 10.8
Insulin (μIU/mL)	8.0 ± 4.5
HOMA-IR	2.0 ± 1.3
Total cholesterol (mg/dL)	240.8 ± 29.7
Log transformed TG (mg/dL)	4.8 ± 0.6
HDL-C (mg/dL)	58.5 ± 17.5
LDL-C (mg/dL)	146.3 ± 19.9
LDL-C/HDL-C	2.7 ± 0.7
Total-C/HDL-C	4.4 ± 1.0
Apo B (mg/dL)	127.1 ± 20.3
Apo A1 (mg/dL)	159.7 ± 23.8
Fatty liver index	33.7 ± 26.8
Nutrition	
Total energy intake (kcal)	1580.9 ± 539.9
K-MEDAS	4.5 ± 1.6

Data are presented as mean ± standard deviation or number (percentage). Abbreviations: BMI, body mass index; WBC, white blood cell; CRP, C-reactive protein; HOMA-IR, homeostatic model assessment of insulin resistance; TG, triglycerides; HDL-C, high-density lipoprotein cholesterol; LDL-C, low-density lipoprotein cholesterol; Apo B, apolipoprotein B; Apo A1, apolipoprotein Al; K-MEDAS, Korean version of the Mediterranean Diet Adherence Screener.

**Table 2:** Diet composition of KMD and control diet.

	KMD	Control	*p*-Value
Energy (kcal/day)	1198.8 ± 296.7	1500.7 ± 437.1	<0.001
Carbohydrate (%)	52.1± 9.3	55.8 ± 11.6	0.021
Fat (%)	30.2 ± 7.8	27.3 ± 9.6	0.032
Protein (%)	19.2 ± 3.3	16.7 ± 4.8	<0.001
MUFA (g/1000 kcal)	13.3 ± 5.7	7.1 ± 4.4	<0.001
PUFA (g/1000 kcal)	7.8 ± 2.9	5.8 ± 2.9	<0.001
SFA (g/1000 kcal)	6.9 ± 3.7	6.8 ± 4.6	0.937
Omega-3 PUFA (g/1000 kcal)	1.2± 0.6	0.7 ± 0.6	<0.001
Omega-6 PUFA (g/1000 kcal)	10.2 ± 2.2	4.9 ± 2.5	0.002
Omega-6/Omega-3	6.13 ±0.49	9.48 ±6.21	<0.001
Fiber (g/1000 kcal)	10.2 ± 4.1	7.7 ± 4.7	<0.001
Trans fat (g/1000 kcal)	0.2 ± 0.2	0.3 ± 0.2	0.004
Cholesterol (mg/1000 kcal)	93.6 ± 72.8	110.4 ± 96.5	0.151
K-MEDAS	9.00 ± 2.26	5.36 ± 2.18	<0.001

Data are presented as mean ± standard deviation or number. Abbreviations: KMD, korean style mediterranean diet, MUFA, monounsaturated fatty acid; PUFA, polyunsaturated fatty acid; SFA, saturated fatty acid; K-MEDAS, Korean version of the Mediterranean Diet Adherence Screener.

## Data Availability

Not applicable.

## Appendix A

**Table A1:** Comparison of changes in metabolic parameters according to diet type.

			Unadjusted			Adjusted		
	KMD (*n* = 92)	Control (*n* = 92)	Sequence *p*-Value	Period *p*-Value	Group *p*-Value	Sequence *p*-Value	Period *p*-Value	Group *p*-Value
Anthropometric parameters								
Weight (kg)	−1.76 (0.14)	−0.26 (0.14)	0.4209	0.6070	<0.001	0.2872	0.9069	<0.001
BMI (kg/m^2^)	−0.63 (0.05)	−0.18 (0.05)	0.6120	0.4410	<0.001	0.5146	0.6475	<0.001
WC (cm)	−1.73 (0.43)	−0.15 (0.43)	0.9539	0.3516	0.0110	0.9662	0.4128	0.1017
Metabolic parameters								
WBC count (no./μL)	−0.40 (0.11)	0.19 (0.11)	0.1049	0.9041	0.0004	0.1686	0.7556	0.0021
CRP (mg/L)	−0.58 (0.58)	0.83 (0.58)	0.8373	0.4798	0.0909	0.8873	0.4987	0.0516
Fasting glucose (mg/dL)	−2.26 (0.72)	1.19 (0.72)	0.7081	0.3540	0.0010	0.7042	0.3676	0.0019
Insulin (μIU/mL)	−1.51 (0.34)	0.33 (0.34)	0.6088	0.3088	0.0002	0.8694	0.1682	0.0182
HOMA-IR	−0.43 (0.09)	0.10 (0.09)	0.5613	0.2027	<0.001	0.8140	0.1080	0.0050
Total cholesterol (mg/dL)	−15.97 (2.74)	−0.93 (2.74)	0.9018	0.0057	0.0002	0.9375	0.0070	0.0012
TG (mg/dL)	−24.35 (8.61)	−0.07 (8.61)	0.0659	0.3322	0.0492	0.0966	0.2277	0.1667
HDL-C(mg/dL)	−3.57 (1.13)	0.41 (1.13)	0.4244	0.0053	0.0149	0.4644	0.0060	0.0184
LDL-C (mg/dL)	−8.73 (2.03)	0.32 (2.03)	0.9602	0.0058	0.0022	0.9774	0.0070	0.0146
Apo B (mg/dL)	−3.83 (3.01)	−3.55 (3.01)	0.5228	0.2023	0.9470	0.5969	0.2838	0.8930
Apo A1 (mg/dL)	0.90 (3.41)	−2.74 (3.41)	0.6855	0.0632	0.4523	0.7932	0.1341	0.4303
Fatty liver index	−8.32 (1.01)	1.23 (1.01)	0.0169	0.3128	<0.001	0.0300	0.5395	<0.001

Data are presented as mean (standard error) in anthropometric and metabolic parameters after each diet. Adjusted for age, sex, total energy intake change, alcohol consumption, smoking status, physical activity change. Sequence *p*-value: KMD-control diet vs. control diet-KMD, period *p*-value: period 1 vs. period 2, group *p*-value: KMD vs. control diet. Abbreviations: BMI, body mass index; WC, waist circumference; WBC, white blood cell; CRP, C-reactive protein; HOMA-IR, homeostatic model assessment of insulin resistance; TG, triglycerides; HDL-C, high-density lipoprotein cholesterol; LDL-C, low-density lipoprotein cholesterol; ApoB, apolipoprotein B; ApoA1, apolipoprotein Al.

**Table A2:** Changes of metabolic parameters according to diet type in both groups.

	Group 1 (*n* = 45)			Group 2 (*n* = 47)		
	Baseline	After KMD	After Control	Baseline	After Control	After KMD
Anthropometric parameters						
Weight (kg)	69.1 ± 14.1	67.2 ± 13.5	66.9 ± 13.6	65.6 ± 11.8	65.4 ± 12.1	63.7 ± 11.8
BMI (kg/m^2^)	25.3 ± 4.4	24.6 ± 4.1	24.4 ± 4.2	24.2 ± 3.1	24.0 ± 3.3	23.4 ± 3.2
WC (cm)	85.9 ± 10.4	84.3 ± 10.7	83.8 ± 10.5	83.2 ± 10.2	82.8 ± 10.3	81.8 ± 10.3
Metabolic parameters						
WBC count (no./μL)	5.4 ± 1.3	5.2 ± 1.1	5.5 ± 1.4	5.6 ± 1.3	5.7 ± 1.3	5.1 ± 1.5
CRP (mg/L)	1.2 ± 2.5	1.0 ± 2.9	1.6 ± 4.1	0.9 ± 0.8	1.9 ± 7.3	0.9 ± 1.4
Fasting glucose (mg/dL)	99.9 ± 8.4	97.9 ± 7.3	98.4 ± 8.2	101.4 ± 12.5	103.3 ± 13.4	100.7 ± 11.9
Insulin (μIU/mL)	8.3 ± 4.5	7.2 ± 4.6	7.4 ± 4.4	7.8 ± 4.5	8.3 ± 4.1	6.4 ± 3.1
HOMA-IR	2.1 ± 1.2	1.8 ± 1.2	1.8 ± 1.1	2.0 ± 1.4	2.2 ± 1.3	1.6 ± 0.9
Total-C (mg/dL)	246.6 ± 34.0	225.4 ± 25.2	230.2 ± 31.4	235.7 ± 24.5	229.3 ± 32.1	218.3 ± 24.4
TG (mg/dL)	134.7 ± 77.2	125.8 ± 111.4	132.1 ± 77.3	160.7 ± 105.1	156.7 ± 103.6	113.6 ± 68.2
HDL-C(mg/dL)	62.5 ± 21.1	56.2 ± 12.4	58.1 ± 14.1	55.0 ± 11.6	53.7 ± 11.4	53.2 ± 9.6
LDL-C (mg/dL)	148.9 ± 21.5	136.2 ± 18.7	140.6 ± 24.7	144.0 ± 18.2	140.4 ± 22.9	135.5 ± 18.5
LDL-C/HDL-C	2.6 ± 0.7	2.5 ± 0.6	2.5 ± 0.7	2.7 ± 0.7	2.7 ± 0.8	2.6 ± 0.6
Total-C/HDL-C	4.2 ± 1.0	4.2 ± 0.8	4.5 ± 1.1	4.5 ± 1.0	4.5 ± 1.1	4.2 ± 0.8
Apo B (mg/dL)	128.5 ± 23.6	117.5 ± 18.5	120.8 ± 20.7	125.9 ± 16.6	123.6 ± 23.9	115.8 ± 17.6
Apo A1 (mg/dL)	163.5 ± 27.3	153.8 ± 22.3	159.9 ± 22.4	156.7 ± 19.7	154.7 ± 16.8	152.9 ± 19.5
Fatty liver index	34.6 ± 27.2	28.0 ± 26.0	30.5 ± 27.2	31.8 ± 26.4	30.5 ± 26.4	21.8 ± 19.4

Data are presented as mean ± standard deviation in anthropometric and metabolic parameters after each diet. Abbreviations: BMI, body mass index; WC, waist circumference; WBC, white blood cell; CRP, C-reactive protein; HOMA-IR, homeostatic model assessment of insulin resistance; Total-C, total cholesterol; TG, triglycerides; HDL-C, high-density lipoprotein cholesterol; LDL-C, low-density lipoprotein cholesterol; Apo B, apolipoprotein B; Apo A1, apolipoprotein Al.

**Table A3:** Comparison of changes in lipid profile after period 1.

	KMD	Control	*p*-Value
Total cholesterol	−21.16 ± 30.08	−6.43 ± 22.81	0.009
Triglyceride	−4.0 (−37.5, 10.0)	−1.0 (−42, 20)	0.817
HDL-cholesterol	−4.0 (−9, 1.0)	−2.0 (−5, 4.0)	0.095
LDL-cholesterol	−12.71 ± 18.06	−3.62 ± 17.29	0.015

Abbreviation: KMD, Korean style Mediterranean diet; HDL, high-density lipoprotein; LDL, low-density lipoprotein.

**Table A4:** Comparison of metabolic parameters according to diet type after adjusting weight reduction change.

	KMD	Control	Sequence *p*-Value	Period *p*-Value	Group *p*-Value
WBC count (no./μL)	−0.31 (0.13)	0.09 (0.13)	0.1166	0.7405	0.0481
CRP (mg/L)	−0.60 (0.67)	0.84 (0.67)	0.8214	0.4940	0.1700
Fasting glucose (mg/dL)	−2.22 (0.83)	1.16 (0.82)	0.7505	0.3655	0.0105
Insulin (μIU/mL)	−0.95 (0.37)	−0.23 (0.37)	0.7139	0.1577	0.2174
HOMA-IR	−0.30 (0.10)	−0.03 (0.10)	0.6710	0.1009	0.0862
Total cholesterol (mg/dL)	−15.12 (3.16)	−1.78 (3.15)	0.8819	0.0072	0.0083
Triglyceride (mg/dL)	−12.40 (9.60)	−11.99 (9.59)	0.0513	0.2086	0.9787
HDL-C (mg/dL)	−3.32 (1.30)	0.17 (1.30)	0.5271	0.0061	0.0901
LDL-C (mg/dL)	−8.40 (2.34)	−0.01 (2.33)	0.9830	0.0071	0.0242
Fatty liver index	−6.28 (1.04)	−0.79 (1.03)	0.0057	0.5397	0.0011
Apo B (mg/dL)	−2.24 (3.41)	−5.15 (3.41)	0.5394	0.2872	0.5866
Apo A1 (mg/dL)	2.27 (3.87)	−4.12 (3.86)	0.7412	0.1361	0.2934

Adjusted for age, sex, weight reduction, total energy intake change, alcohol consumption, smoking status, physical activity change. Abbreviation: WBC, white blood cell; CRP, C-reactive protein; HOMA-IR, homeostatic model assessment of insulin resistance; HDL-C, high-density lipoprotein cholesterol; LDL-C, low-density lipoprotein cholesterol; Apo B, apolipoprotein B; Apo A1, apolipoprotein Al.
